# Surface Characterization of Metallic Materials in the Case of the Turning Process of NiTi Alloy

**DOI:** 10.3390/ma17020487

**Published:** 2024-01-19

**Authors:** Anna Zawada-Tomkiewicz, Dariusz Tomkiewicz

**Affiliations:** Faculty of Mechanical Engineering, Koszalin University of Technology, 2 Śniadeckich Str., 75-453 Koszalin, Poland; dariusz.tomkiewicz@tu.koszalin.pl

**Keywords:** surface roughness, PCA, Hilbert–Huang transform, minimal undeformed chip thickness

## Abstract

A study was made of the machinability of NiTi alloy in turning, under conditions resulting in a small cutting layer. The experiment involved cutting with variable feeds ranging from 0.01 to 0.1 mm/rev. The cutting conditions were carefully chosen, considering the rounding radius of the cutting edge. The machined surface was examined and measured in 3D using a confocal microscope and in 2D with a contact profilometer. These measurements were used to estimate *h_min_*, leading to the development of a surface formation model that considers both the lateral material flow due to *h_min_* and the lateral material flow due to altered thermodynamic conditions from the previous blade pass. A method for evaluating the surface and selecting its characteristics was proposed based on analyses derived directly from surface features: PCA (Principal Component Analysis) and EMD (Empirical Mode Decomposition) with the Hilbert transform (Hilbert–Huang transform). PCA analysis facilitated the examination of individual surface component variances, while analysis of the IMF components enabled the assessment of surface component energy combined with instantaneous frequencies.

## 1. Introduction

### 1.1. Machinability of NiTi Alloy

The machinability of the material used in cutting tests, including the NiTi alloy, is a concept that requires a comprehensive approach [1]. Critical factors relating to the material are its chemical composition, structural properties, and the uniform distribution of those properties throughout the volume of the material being cut. Machinability is influenced by the variables of the cutting process, which is the result of the interaction of individual elements of the machining system, in particular the machine tool, the tool, and the workpiece. The machinability of the material is determined by the multi-criteria optimization of the cutting parameters [2].

Existing knowledge relating to the machining of NiTi alloys indicates significant interest in these materials, although there is a relatively limited number of studies specifically addressing machining aspects. NiTi alloys have been studied since the 1960s, and research has primarily focused on their thermo-mechanical properties [3,4]. Despite the thorough exploration of these alloys in various scientific contexts, a noticeable gap exists in the dedicated study of their machining characteristics. This underscores the need for further investigation in this specific area.

NiTi alloys display various distinctive attributes, including biocompatibility, remarkable ductility, a high strength-to-weight ratio, good resistance to fatigue and corrosion, and notable damping capacities [5]. Although these features make them excellent for various applications, the cutting of NiTi alloys poses challenges due to their pronounced ductility, sensitivity to temperature, and the tendency for strong local material hardening.

In the machining of NiTi alloys, a significant amount of material often remains attached to the workpiece, appearing as undesirable material side flow [6,7]. In recent years, there has been a noticeable effort to explore the feasibility of processing NiTi alloys using conventional machining methods. Despite the challenges caused by their inherent characteristics, these efforts signify a growing interest in developing effective techniques for machining NiTi alloys. The challenges identified in the machining of NiTi alloys, as presented in [8], include concerns about the quality of the machined surface, in addition to the issue of rapid tool wear. The observed adverse physical phenomena in the machining of these challenging materials arise from their distinctive properties, such as super-elasticity and phase changes during the machining process.

In finishing operations, the machining process becomes even more intricate, due to the size effect. This complexity contributes to material springback in ductile phases and introduces ploughing effects when the undeformed chip thickness aligns with or falls below the cutting-edge radius [9]. These complexities highlight the intricacy of the machining of NiTi alloys, which requires a nuanced approach to address the specific challenges resulting from their physical characteristics.

### 1.2. Motivation and Research Objectives

As mentioned above, the machining potential of NiTi alloys is limited due to their low and variable modulus of elasticity for different phases, coupled with other material properties, which cause them to be categorized as difficult-to-machine materials [6].

This article presents a multi-aspect approach to testing the machinability of the material in turning tests where the cut layer has a small cross-section, in the context of surface roughness formation. A detailed analysis is made of surface roughness at various feed rates in the dry turning of the NiTi alloy. An initially treated NiTi surface was subjected to detailed measurements and 3D analysis. The use of PCA analysis helped in the classification of the measured surfaces, enabling the application of an appropriate analytical workflow.

The machined surface was also measured and analyzed in a 2D system, which is particularly suitable for turned surfaces. The test includes a parametric analysis of surface roughness, providing a comprehensive assessment of the profile of the machined surface. Such analysis is crucial considering the various effects occurring during the cutting of NiTi alloys, which can have an adverse effect on surface roughness. The Hilbert–Huang transform was used for the surface profile.

## 2. Materials and Methods

The objective of this study was to evaluate the surface roughness achieved in a turning process using coated sintered carbide tools. Turning tests were conducted on a high-rigidity CNC Gildemeister NEF 400 lathe. A 90 mm-long annealed NiTi alloy rod [10] with an external diameter of 18 mm was used (Wolften Ltd., Wroclaw, Poland). The material specifications are summarized in Table 1. The composition of the material was confirmed using a Phenom G2 Pro scanning microscope (EDS) and on a TGA/DSC/FTIR Netzsch thermal analyzer (DSC).

In research efforts, considerable emphasis has been placed on describing NiTi alloy compositions, but studies related to the machining of such alloys are quite limited. In one study [11], coated carbide inserts were utilized in turning trials. The investigation considered three cutting speeds and two feed rates with a cutting depth of 0.5 mm. It led to a recommended cutting speed of 25 m/min, with a feed rate of 0.1 mm/rev, in the case of both dry cutting and minimal lubrication.

In another study [12], under similar cutting conditions, it was determined that the lowest wear during dry cutting occurred at a cutting speed of 12.5 m/min, while the lowest cutting forces were observed at a cutting speed of 25 m/min. In independent research [13], where a range of cutting speeds from 12.5 to 75 m/min was analyzed (feed rate = 0.1 mm/rev, depth of cut = 0.3 mm, dry), the minimum tool wear on both the major flank B (VBB in accordance with [14]—width of the abrasive major flank wear) and in the area of the rounded C corner (VBC in accordance with [14]—width of the abrasive wear in the area of the blade corner C) was observed at a cutting speed of 12.5 m/min. However, surface roughness tests for the specified cutting conditions indicated that increasing the cutting speed to 25 m/min resulted in a significantly smoother surface. Therefore, a cutting speed of 25 m/min was selected for this study.

The primary objective is to apply finishing operations, where constraints relate to the depth of cut and the feed rate, with a limiting value of 0.1 mm/rev. An experiment was designed using a cutting tool in the form of a cemented carbide insert coated with a PVD layer (physical vapor deposition layer), (Ti,Al)N + TiN, mounted in a turning holder with the symbol DWLNR-2020-K08.

The values for the depth of cut and feed rate were set at 0.3 mm to ensure that the cutting process would occur, facilitating chip formation. The selection of the feed rate and depth of cut took into account the radius of the cutting-edge rounding. The methodology for determining the radius of the cutting-edge rounding has been detailed in various studies [15]. It involves the selection of measurement equipment capable of non-destructively capturing a cross-section perpendicular to the edge. Contact and optical profilometers, interferometers, confocal microscopes, and metallographic microscopes are commonly employed for this purpose [16].

**Figure 1:**
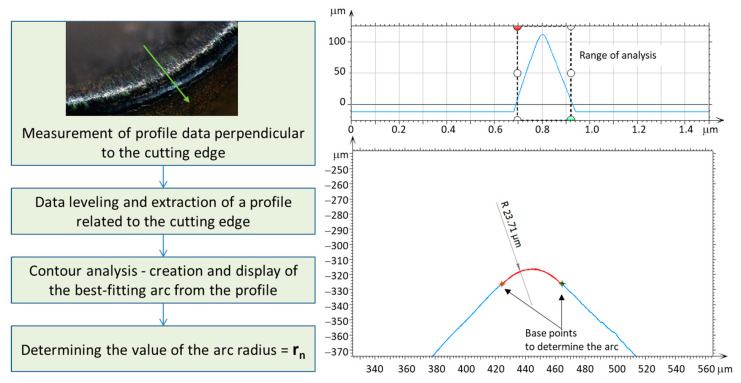
Algorithm for determining the value of the tool edge radius.

For the identified cross-section of the cutting edge, geometric characteristics are determined to inscribe a circle, with its radius representing the radius of the cutting-edge rounding. An example determination of the radius of the cutting-edge rounding is illustrated in Figure 1. Using data collected from the contact profilometer, an arc was plotted, the radius of which was measured repeatedly for each blade to determine the value of the cutting-edge rounding radius. The value of the cutting-edge rounding radius for different inserts ranged from 18 μm (minimum value) to 25 μm (maximum value).

Employing experimental design principles, the feed rate was selected in a manner that ensured that a portion of the test was conducted under extremely unfavorable conditions (micro-cutting) [17]. In these conditions, there was a high probability that the feed rate was below the radius of the cutting-edge rounding. The adverse conditions also included the continuous wear of the tool during cutting and the tendency for an increase in the radius of the cutting-edge rounding (the edge becoming duller). Table 2 lists the cutting parameters.

## 3. Surface Measurement and Discussion

The fundamental objective of measurement was to collect data on the surface texture in both 2D and 3D perspectives. The assessment of surface roughness in machining operations is crucial, as it determines properties such as the visual appearance of the texture, friction, and wear [18]. In general, a smaller roughness value corresponds to a smoother and more favorable surface [19].

In this study, the measurements of surface roughness were conducted in both 2D and 3D perspectives. Three-dimensional measurements pertain to the surface and are referred to as topography or stereometry measurements. A confocal laser scanning microscope (Olympus LEXT OLS4000) with a magnification of ×10 was used, and topographic data of 1280 × 1280 μm were recorded. Two-dimensional measurements focused on the surface profile and irregularities within a designated section of the profile, and were carried out using a Hommelwerke T8000 stylus profilometer with TURBO Roughness for Windows software. Independently, 24 measurements were performed for each measurement point. The data in both 2D and 3D perspectives were processed using MathWorks^®^ Matlab^®^ ver. 2023b and Digital Surf MountainsMap^®^ ver. 10 software.

Parametric analysis of the surface is typically conducted in accordance with the ISO 25178 series [20] for 3D analysis or ISO 21920 series [21] for 2D analysis. It includes considerations such as the selection of signal pre-processing methods, filtration methods, cut-off, and the selection of surface parameters from a set of possibilities. The evaluation takes into account factors such as classification bias, dispersion of values, and measurement capabilities.

**Figure 2:**
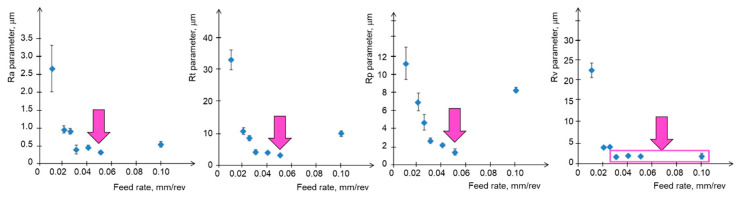
Surface profile height parameters as a function of feed rate with an indication of the most favorable values.

The surface roughness analyses for turning operations focus on the kinematic-geometrical mapping of the tool nose. The radius of the tool nose determines an almost flat stretch parallel to the surface, with a length similar to the value of the feed rate, especially in the case of micro-machining. The theoretical maximum roughness, calculated for cutting conditions, is estimated to be 3.1 µm (f = 0.1 mm/rev). The measured roughness was slightly higher than the theoretical roughness for larger feed rates, and significantly higher for smaller feed rates—the smaller the feed rate, the less the kinematic-geometric model reflects the process of micro-roughness formation on the surface. This is due to the scale effect and material properties. The results for the determined height parameters of the surface—Ra, Rt, Rp, and Rv—are presented in Figure 2, which indicates the points with the lowest roughness.

**Figure 3:**
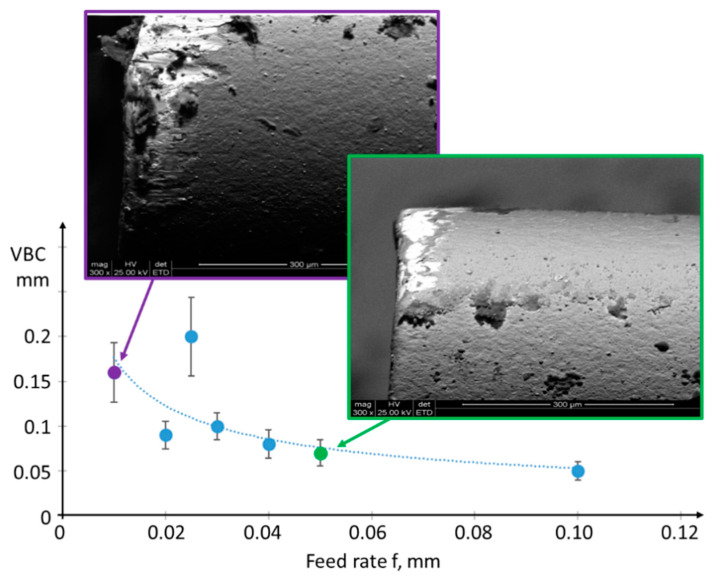
The wear value of the cutting edge VBC (in accordance with [14]—width of the abrasive wear in the area of the blade corner C), along with sample SEM images of the tool minor flank surface, captured at a magnification of ×600 (FEI Quanta 200 Mark II).

The increased surface roughness compared with the theoretical model is mainly due to the poor thermal conductivity of the NiTi alloy, which prevents the sufficient removal of heat from the machining zone [22]. This is evident in the continuous accelerated wear of the tool and the blunting of the cutting edge (Figure 3). Machining with a feed rate of 0.01 mm/rev instead of 0.1 mm/rev results in a significantly smaller cross-sectional layer being cut, but a ten times longer cutting time, and consequently a ten times longer contact time between the tool and the workpiece. This leads to a higher specific cutting pressure and a greater amount of generated heat.

The emergence of tool wear affects the minimal undeformed chip thickness. It follows that an increase in the radius of the blade’s edge and blunting of the cutting edge increases the effect of the side flow of the material, consequently increasing the amplitude of surface height at low frequencies. Additionally, tool wear is associated with cutting edge chipping, with the envelope reproducing in the range of the high spatial frequencies of the surface. Cutting with worn edges, featuring a non-zero edge radius under constrained cutting conditions, causes an increase in roughness [23].

As is evident from the measurement results, the quality of the machined surface correlates with numerous process variables, and the surface is a product of the mechanical machining process and its conditions. Developing a suitable model for the machined surface in the cutting process is a non-trivial problem due to the multitude of factors influencing the machined surface. Predicting outcomes in such complex conditions is nearly impossible.

The main approaches to developing a model focus on the theory of cutting, experimental modeling, and, more recently, artificial intelligence [24]. The theory of cutting is applied in situations where there is a comprehensive understanding of the process. This requires knowledge of the process kinematics, cutting tool and material properties, chip formation mechanism, and numerous other factors. Since full knowledge of the cutting process is not always available, the experimental approach often employs modeling methodologies. However, it should be noted that the results of experimental research have limited overall applicability. To enhance the predictability of surface roughness in an experimental approach, a systematic method is employed during experiment planning, data collection, and analysis.

To overcome the limitations of the above methods and leverage their advantages simultaneously, artificial intelligence methods have been developed. These methods allow the integration of signals from different sensors, handling incomplete knowledge through fuzzy inference, and incorporating task descriptions. Surface measurement data and simulations of theoretical models based on cutting parameters, chip formation, cutting forces, and other components are utilized to monitor surface quality. 

The relationship between surface characteristics and the conditions (parameters) of the technological process is expressed in the form of a surface constitutive function. The fundamental model for generating surface roughness in turning is the kinematic-geometric model ($f^{2}/{8r}_{\varepsilon }$). This model holds theoretical significance and serves as the foundational model for others developed by different authors [25,26,27] who have analyzed the scale effect and augmented the model with additional components to approximate it to real machining conditions. However, it should be noted that these considerations are based on the concept of the minimal undeformed chip thickness, $h_{min}$.

**Figure 4:**
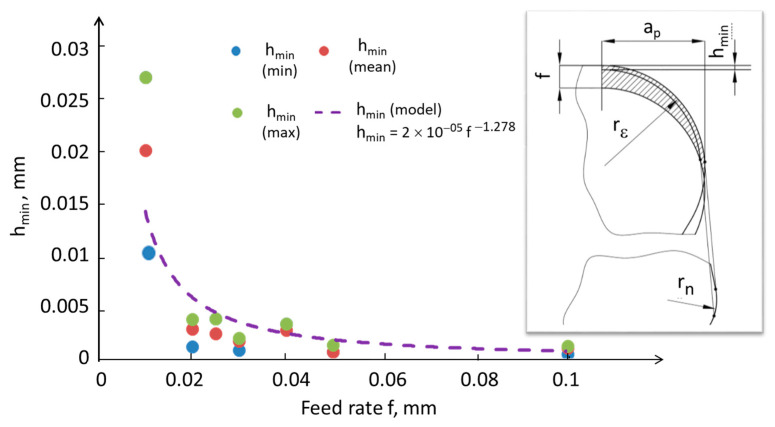
Minimum undeformed chip thickness ($h_{min}$) values as a function of feed rate, along with the definition of $h_{min}$ (f—feed rate, $a_{p}$—depth of cut, $r_{\varepsilon }$—tool nose radius, $r_{n}$—radius of tool edge rounding).

Determining the probable minimum thickness of the machined layer based on simplified material and geometric assumptions, preceded and supplemented by experiments relating to the fundamentals of cutting, makes it possible to capture the stochastic nature of chip separation phenomena. The minimum thickness of the machined layer is closely dependent on machining conditions, including the properties of the workpiece material and the tool material and geometry. Figure 4 presents a model of the machining process with an emphasis on the cross-section of the machined layer. When a chip appears, the phenomenon of decohesion occurs in the area representing a fraction of the edge radius value $r_{n}$. A significant challenge in modeling is the fact that, contrary to the model’s assumptions, the transition from the elastic stress of the cutting-edge interaction to the volume of the removed chip does not occur in a single plane, but in a defined volume of material. In this case, the scale effect is noticeable. The $h_{min}$ value was determined on the basis of the experiment (roughness value obtained for 30 repetitions) and the model inverse to the so-called Brammertz formula [28]. The value of $h_{min}$ estimated based on the experiment was very small, below 1 μm for larger feeds and not exceeding 4 μm for smaller feeds (except in the experiment with a feed of 0.01 mm/rev, for which $h_{min}$ averaged over 10 μm and in some cases even 20 μm).

**Figure 5:**
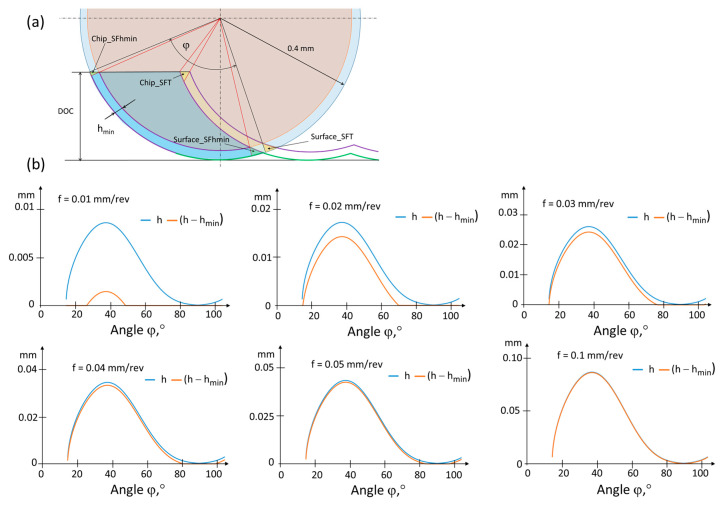
(**a**) Cross-section of the machined layer with highlighted areas of material side flow based on $h_{min}$, referring to $Surface\_SFh_{min}$ and $Chip\_SFh_{min}$, as well as due to elevated temperature, specifically Surface_SFT and Chip_SFT; (**b**) theoretical cross-section of the machined layer (h) and the cross-section of the machined layer after considering $h_{min}$ as a function of the angle φ.

In Figure 5a, the geometric cross-section of the machined layer is depicted, highlighting the minimum thickness of the machined layer. Simultaneously, in Figure 5b, the theoretical thicknesses of the machined layer are graphically represented as a function of the angle *φ*, both considering and not considering the minimal undeformed chip thickness $h_{min}$. It was observed that for smaller feeds, after considering the calculated value of $h_{min}$ based on the model from Figure 4, the area in which material removal is difficult to cut is relatively large, and depending on the local thermodynamic properties in the machining zone [25], the material will tend to undergo side flow.

**Figure 6:**
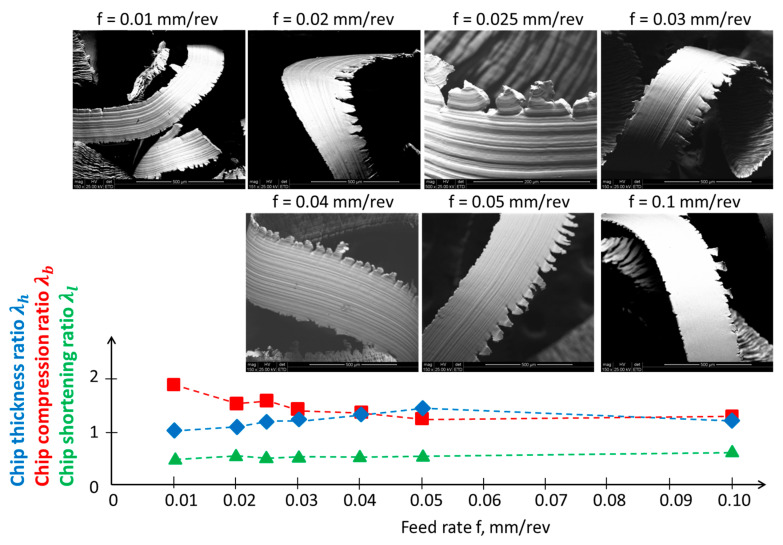
Analysis of chip compression ratios, with sample SEM images of chips collected for each experimental point (FEI Quanta 200 Mark II).

According to Figure 5a, the cross-section of the machined layer with highlighted areas of the material side flow based on $h_{min}$—referring to $Surface\_SFh_{min}$ and $Chip\_SFh_{min}$—will deform freely. However, the consequence of a non-zero value of $h_{min}$ on the machined surface side is that cutting takes place under different thermodynamic conditions, significantly higher temperatures, and altered material properties compared with the previous tool pass. This lateral material flow is indicated in Figure 4 as Surface_SFT for the surface and Chip_SFT for the chip. The deformation of the material in the form of the side flow on the chip surface is illustrated in Figure 6.

The assessment of the impact of the feed on the chip shape, based on [14], allows the conclusion that with an increase in the feed, the chip shape becomes more uniform and changes to a continuous ribbon form; it also becomes an unfavorable entangled chip. The chip compression ratios indicate that the chip becomes thicker as the feed increases. This means that for a feed greater than the radius of the cutting-edge rounding, there are favorable conditions for chip formation. According to the methodology presented in [29], for constant speed and material diffusivity [30], the Peclet number is estimated to be small, below 2, which means that the plastic deformation of the chip takes place under conditions of high thermal energy input.

**Figure 7:**
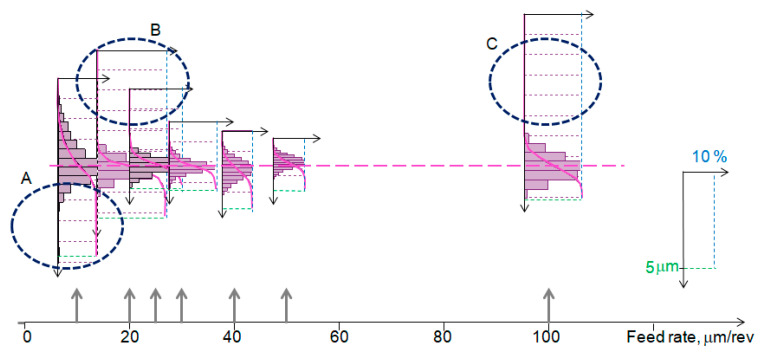
Material ratio function for variable feed rate.

**Figure 8:**
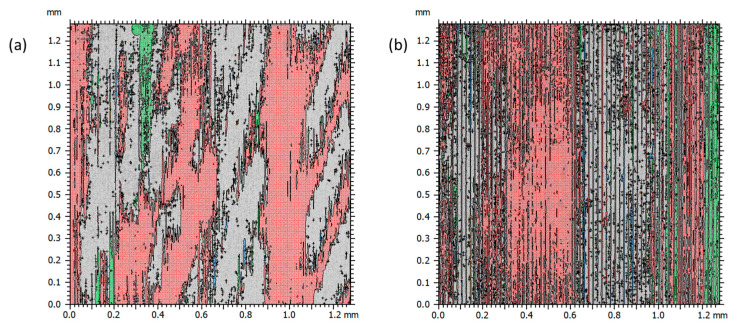
Surface map highlighting areas above the 50% threshold for feed rate (**a**) 0.01 mm/rev and (**b**) 0.02 mm/rev.

The influence of the feed rate on the functional properties of the surface can be illustrated using the material ratio function, plotted on the same scale in Figure 7. Region A indicates the presence of deep and large valleys for feed rates smaller than 0.01 and 0.02 mm/rev. In Figure 8, areas in the surface above the 50% threshold are highlighted in color. For a feed rate of 0.01 mm/rev, surface vibrations were observed, and both surfaces are characterized by strong variation and barely noticeable traces of feed. Regions B and C indicate the occurrence of locally large individual protrusions.

**Figure 9:**
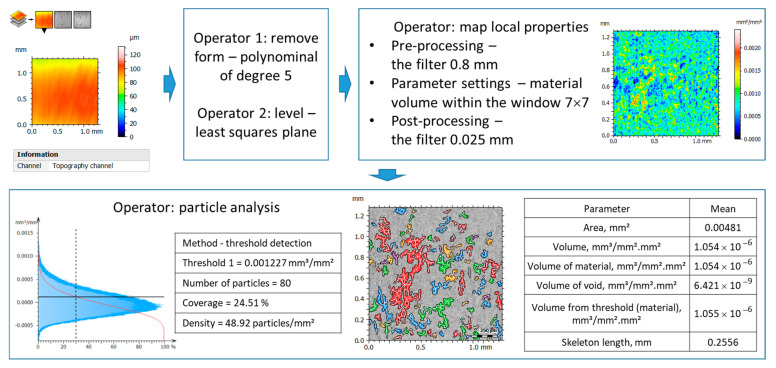
Algorithm for analyzing local properties of surface topography.

The analysis of the influence of the feed rate, and thus $h_{min}$, on the geometric structure of the surface was also visualized through the analysis of the local surface topography properties in a 3D context. Figure 9 presents the algorithm for analyzing the local properties of surface topography along with an example for a feed rate of 0.01 mm/rev. The algorithm consists of the following main stages: preparation of data for analysis, local mapping of properties, and analysis of the volume of material within a specified space (above the defined threshold).

**Figure 10:**
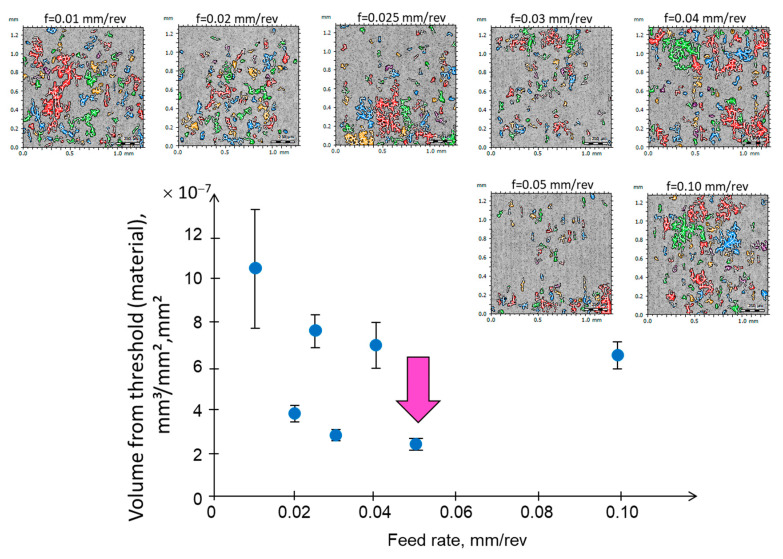
Analysis of the volume of surface material located above the defined threshold (30% according to the algorithm in Figure 9) for various feed rate values.

From the analysis of the volume of the surface material located above the defined threshold for various feed rate values, it follows that if the volume of material in a certain microvolume is analyzed, the phenomenon of lateral material flow can be observed for each cutting trial (Figure 10). The phenomenon of lateral material flow is more pronounced at smaller feed rates. The classification of microvolumes based on their orientation indicates the prevalence of orientations aligned with the feed direction. The orientation of the lateral side flow occurs in various directions. In the NiTi cutting process, several other factors influence surface roughness besides the feed rate. These factors include the shape memory effect and superelasticity. When combined with the low thermal conductivity and ductility of the material, they affect the chip breaking process and the formation of both $h_{min}$ side flow and temperature side flow. All of these factors negatively impact surface texture, with deviation from the model resulting from the kinematics of the process.

## 4. Results and Discussion on Surface Characterization

**Figure 11:**
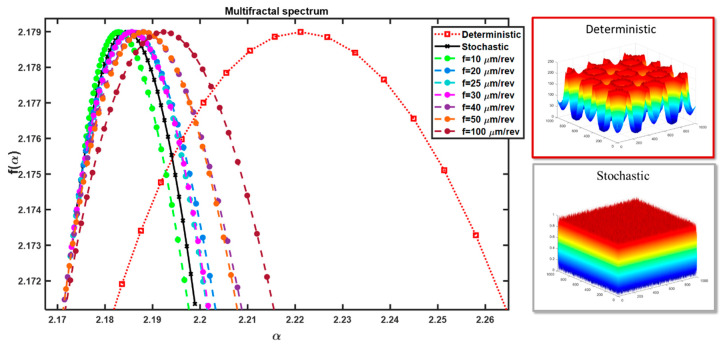
Multifractal spectrum of the surface after turning of NiTi alloy, for cutting speed $v_{c}$ = 25 m/min and various feed rates.

To provide a thorough characterization of the surface, an initial attempt was made to assess its nature. For this purpose, multifractal analysis was employed based on the methodology outlined in [31]. This involves the distribution of the singularity spectrum f(α) as a function of the singularity exponent α (fractal dimension). A multifractal analysis in a 3D perspective was conducted for the surface, and the resulting spectrum is depicted in Figure 11.

As observed in Figure 11, all of the spectra obtained fall within a narrow range. Two additional analyses are included on the chart: for a stochastic surface (randomly generated) and a deterministic surface (composed of two-dimensional sinusoidal functions). For the stochastic surface, the exponent α changes within a small range, similar to its distribution. On the other hand, for the surface with a dominant deterministic character, this exponent has a larger range of variability, and the scatter of values in the roughness space is much greater. The multifractal spectrum distribution for the surfaces indicates that they have a stochastic-deterministic character, with a tendency towards stochastic dominance. The surface machined with a feed rate of 0.01 mm/rev clearly exhibits a stochastic character, while that obtained with a feed rate of 0.1 mm/rev deviates significantly towards a deterministic surface and exhibits a pronounced stochastic-deterministic character. 

**Figure 12:**
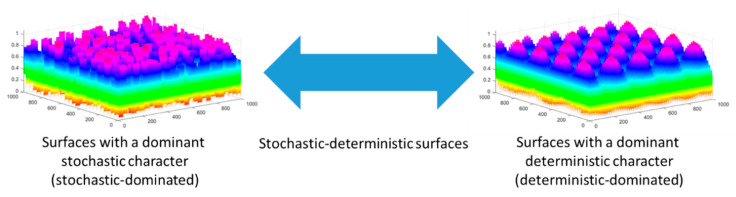
Spectrum of surface features [32].

According to the concept proposed in [32], describing “feature spectra” as shown in Figure 12, which serve as a support system for selecting the method of surface characterization, surfaces with a stochastic-deterministic character should be characterized in both 2D and 3D perspectives. The results are assumed to depend on the measurement location—hence the recommendations for multiple repetitions and statistical analysis.

**Figure 13:**
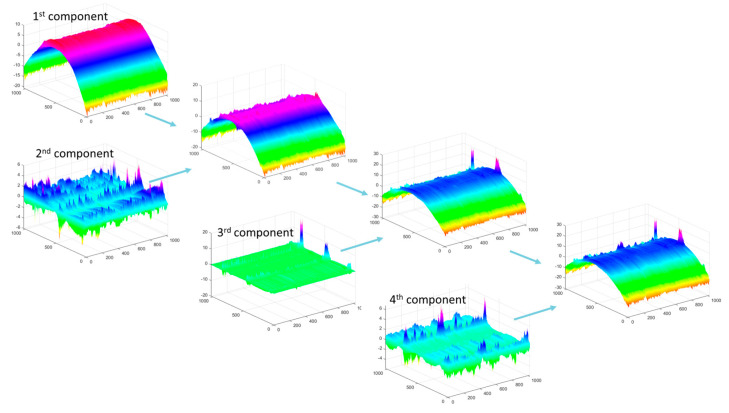
PCA for the first four components for the data presented in Figure 14 IV (f = 0.1 mm/rev).

**Figure 14:**
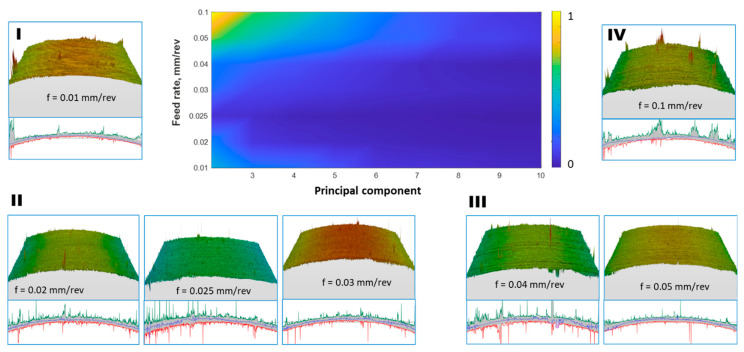
Percentage of the total variance explained by each principal component from the 2nd to 10th components, and visualization of the surface data used for the analysis along with the designation of surface groups I–IV.

**Figure 15:**
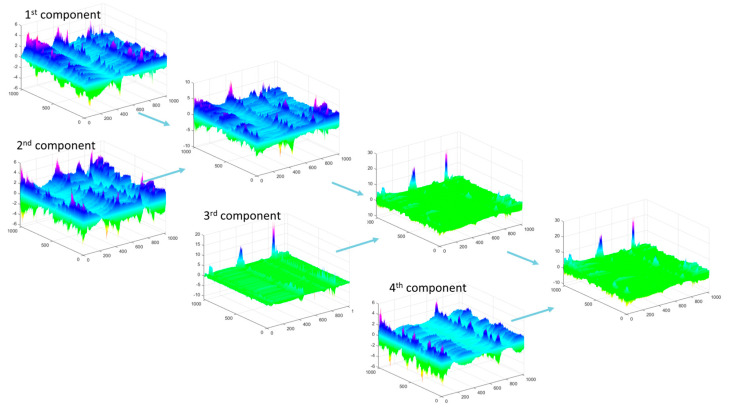
PCA for the first four components for the data presented in Figure 16 IV (f = 0.1 mm/rev).

**Figure 16:**
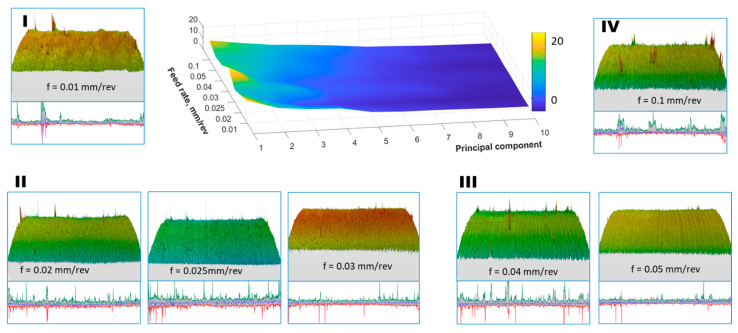
Percentage of the total variance explained by each principal component for the 1st to 10th components, and visualization of surface data used for the analysis (perpendicular to the data used in Figure 14) along with the designation of surface groups I–IV.

Principal Component Analysis (PCA) applied to surface analysis is a method that allows a better understanding of the nature of surface measurement data. Due to its factor analysis nature, PCA decomposes spatial surface data into components depending on what we assume as the basis [33]. It is possible to analyze a set of profiles parallel to the machining traces (Figure 13 and Figure 14) or perpendicular to the machining traces (Figure 15 and Figure 16). This means that, concerning the determined average profile, we examine to what extent spatial surface data vary relative to this mean value.

In the PCA analysis, the Singular Value Decomposition (SVD) algorithm was employed. The first component illustrates data organized into a structure representing the greatest variance and heterogeneity. In subsequent vectors of the matrix, one can identify averaged changes in the profile along the direction parallel to the texture orientation of the surface. The components are elements of the eigenvectors of the surface variance matrix. The first eigenvector indicates the direction in which the data vectors collectively exhibit the greatest variability. A new coordinate system is formed, with each axis aligned along the direction of maximum common variability.

Figure 13 shows the first four components of the PCA distribution for the raw spatial data of machined surfaces with a feed rate of 0.1 mm/rev, collected using the Olympus LEXT OLS4000 laser confocal microscope. As observed, the dominant structure of the surface curvature (first component) is complemented by subsequent components with low variance.

For the variance data provided in Figure 14, after calculations, the first component for spatial data represents over 99% of the total signal energy for all feed rates. Therefore, on the energy map, the maximum value for the second and subsequent components does not exceed 1. The main component 1 reproduces the rounded shape of the cylindrical surface, and the subsequent ones add disturbances to this shape, as these are surface elements of less importance.

Spatial raw data from the surfaces after turning with a feed rate of 0.1 mm/rev were analyzed perpendicular to the machining traces. In Figure 15, the first four components of the PCA distribution are presented. As observed, the first component of surface irregularities is complemented by subsequent components with variance only slightly smaller than the first. In this direction, the signal energy is not concentrated in one component, but rather in the entire group of the first components.

For the energy data presented in Figure 16 (in the 3D plot), the maximum value for the first, second, and subsequent components does not exceed 20. The PCA components correspond to the shape of the basic profile of surface roughness, starting with the component with the highest energy and followed by additional components. For the PCA distribution in this direction, the reconstruction of the original data (>95% signal energy) required several dozen components for each feed rate, with the fewest components needed for a feed rate of 0.1 mm/rev. The PCA analysis allowed the identification of four groups of surfaces, conventionally marked in Figure 14 and Figure 16 from I to IV, with different energy distribution. Group I refers to the surface whose variability is large in both directions of analysis and is divided into many components. It was observed that the larger the group number, the greater the energy concentration on several main components of the PCA decomposition.

Completing the surface characterization with interpretive capabilities regarding the physical components of the machining process, surface roughness analyses based on surface features are created—analysis of spatial-temporal frequency. The analysis process consists of two key stages: creating an EMD (Empirical Mode Decomposition) and applying the Hilbert transform to the components of the EMD distribution (Hilbert–Huang Transform) [34].

**Figure 17:**
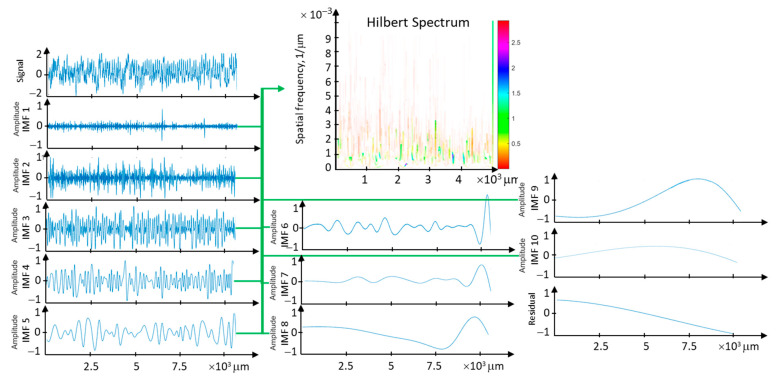
Distribution of EMD data for the surface profile with a feed rate of f = 0.1 mm/rev, and the complete Hilbert spectrum for all 10 IMFs.

The creation of an EMD (Empirical Mode Decomposition) is based on decomposing the input signal into a finite and small sum of components. These components are called the IMF (Intrinsic Mode Function) and are obtained from the signal by applying a sifting algorithm. The procedure is subject to two constraints: the first is that each IMF has the same number of zero-crossings and extrema, while the second constraint imposes symmetry on each IMF function around the mean value. The component with the highest frequency is determined locally by creating upper and lower envelope functions that interpolate the local maxima and minima of the signal. By averaging these envelopes, a signal representing the local mean of the signal is created. Subtracting this mean from the signal results in a signal with local zero-crossings. The first IMF component is created in this way. The remainder after subtracting the first IMF from the signal is treated similarly, and the second IMF component is created, then the third, and so on. This process results in the decomposition shown in Figure 17.

The IMF components of the surface profile have different energy levels. For the surface profile, the energy is concentrated in the kinematic-geometric range of tool mapping, especially if low-frequency phenomena occur in this range. To analyze the energy from individual IMFs, a complete EMD decomposition is performed, followed by the Hilbert transform for each IMF component.

The Hilbert–Huang Transform for the rolling profile data is embedded in the length of the measurement segment. The horizontal axis represents the measurement segment (measurement points, time), and the vertical axis represents the spatial frequency range (Hz). The instantaneous amplitude is presented as a pseudocolor map. The Hilbert transform is analogous to the Fourier power spectral density, but it is local in the time (length) domain. It serves as a measure of the energy carried by the surface profile data for a specific measurement point where the signal oscillates with instantaneous spatial frequency. 

**Figure 18:**
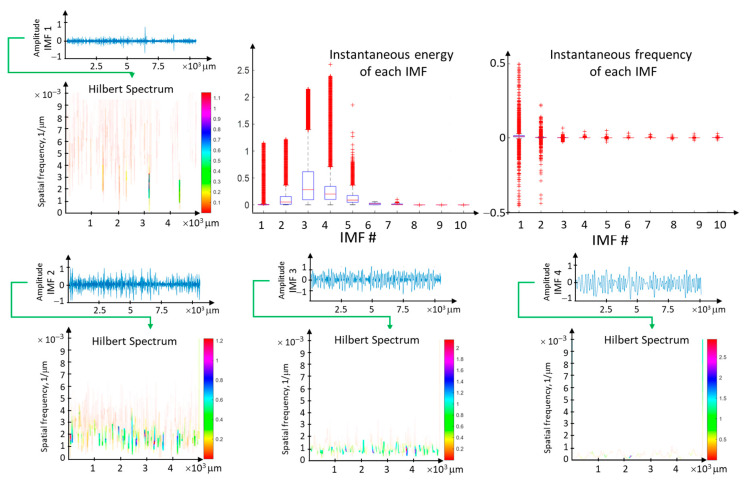
The analysis of the four initial Intrinsic Mode Functions (IMF) of the surface profile data for feed rate f = 0.1 mm/rev in the context of instantaneous frequencies and energy.

The analysis of the four initial Intrinsic Mode Functions (IMFs) of the surface profile data for the feed rate f = 0.1 mm/rev is presented in the context of instantaneous frequencies and energy (Figure 18). The most concentrated energy is observed in the first IMF component within the frequency range resulting from the kinematic-geometric representation of the tool edge after scaling. Higher frequencies correspond to high-frequency disturbances related to lateral material flow, while lower frequencies arise from instabilities in the machining process. The range of instantaneous frequencies is widest for the first component. However, the associated energy is relatively small.

**Figure 19:**
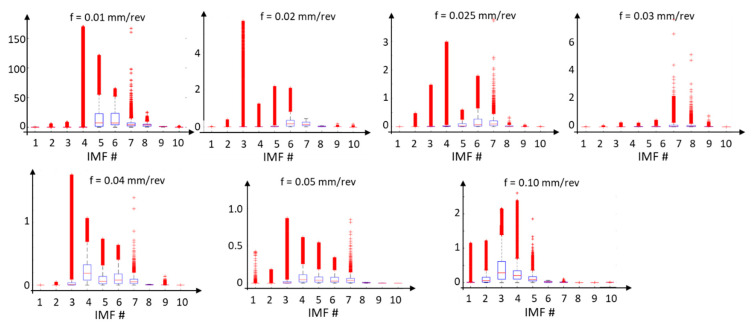
The energy of individual IMFs from the EMD distribution for surface profile data obtained for various feed rates.

**Figure 20:**
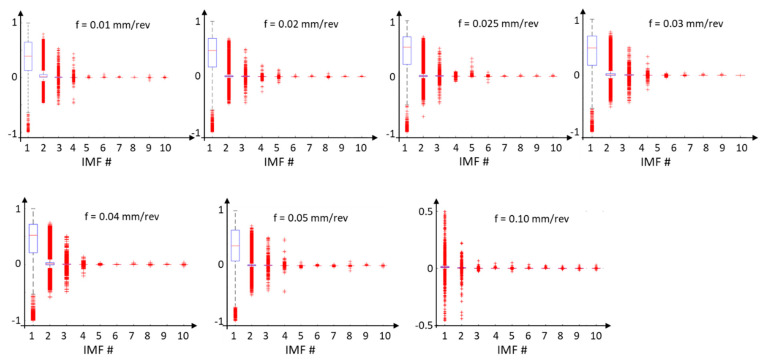
Instantaneous frequencies of surface profile data for different feed rates, with a decomposition of the distribution into individual IMFs from the EMD distribution.

The energy (Figure 19) and instantaneous frequencies (Figure 20) of the surface profile data obtained for various feed rates allow us to trace how the energy is concentrated for individual IMFs from the EMD distribution. For surface profile data with a feed rate of 0.01 mm/rev, the energy is concentrated in IMF 5 and IMF 6. For these components, the dispersion of instantaneous frequencies is small, confirming the presence of vibration in the system. On the other hand, for feed rates of 0.04 mm/rev and 0.05 mm/rev, the signal energy is distributed across several IMFs, indicating a more deterministic signal.

## 5. Conclusions

This study has investigated the process of the cutting of NiTi alloy under conditions of small undeformed chip thickness, where the radius of the cutting edge plays a crucial role. Through experimental studies, the value of $h_{min}$ was determined and subsequently employed to model the scale effect. Lateral side flow was observed on both the surface and chip, both originating from the undeformed chip thickness $h_{min}$ and caused by the material not collected in the preceding pass, which is exposed to high temperatures and pressure.

1. The phenomenon of lateral material flow occurred in every analyzed cutting case. This phenomenon intensified for small feed rates, where a significant portion of the cross-section of the machined layer fell within the region of hindered chip formation (the area between the theoretical nominal cross-section of the machined layer and the cross-section determined experimentally). Lateral material flow was distinguished for the chip, observed in SEM images for each feed rate, as well as lateral material flow for the surface, which required for confirmation the application of various methods for analyzing the surface topography data in both 2D and 3D perspectives.
2. The substantive accuracy of surface characterization is a prerequisite for its effective utilization in an integrated design-manufacturing-use system. To achieve this, it is essential to define the surface characteristics along with the measurement methods for the associated quantities [35]. Multifractal analysis was applied to evaluate the surfaces, revealing predominantly stochastic-deterministic features and enabling the proper selection of surface analysis methods and characteristics. PCA analysis allowed an assessment of the variance of individual components along and across the machining traces for spatial surface data. The use of EMD and the Hilbert transform enabled the determination of signal energy in connection with instantaneous frequencies. The proposed methods were used to conduct analyses solely based on surface features: PCA relies on eigenvalues and EMD analysis results from the envelope of surface data as a time-varying signal. These analyses provided a deeper insight into the surface texture created in the turning of NiTi alloy.

## Figures and Tables

**Table 1:** Specification of the material used for machining.

Material	Nitinol, NiTi alloy
Specification	ASTM F2063
Tensile strength	875 MPa
Elongation	16%
ASTM F2063	nominally 54.5–57.0 wt.% nickel as used for the manufacture of medical devices and surgical implants
ISO material group	S

**Table 2:** Cutting conditions.

Cutting Speed $v_{c}$, m/min	Feed Rate f, mm/rev	Depth of Cut DOC, mm	Cutting Distance, mm
25.0	0.01; 0.02; 0.025; 0.03; 0.04; 0.05; 0.10	0.3	15

## Data Availability

Data are contained within the article.
